# Understanding social needs screening and demographic data collection in primary care practices serving Maryland Medicare patients

**DOI:** 10.1186/s12913-024-10948-7

**Published:** 2024-04-10

**Authors:** Claire M. Starling, Marjanna Smith, Sadaf Kazi, Arianna Milicia, Rachel Grisham, Emily Gruber, Joseph Blumenthal, Hannah Arem

**Affiliations:** 1 Implementation Science, Healthcare Delivery Research Program, MedStar Health Research Institute, 6525 Belcrest Road, Suite 700, Hyattsville, MD 20782 USA; 2 Department of Emergency Medicine, Georgetown University School of Medicine, 3900 Reservoir Road, Washington, NWDC 20007 USA; 3 National Center for Human Factors in Healthcare, MedStar Health Research Institute, 3007 Tilden St.Suite 6N, Washington, NWDC 20008 USA; 4 Maryland Primary Care Program, Maryland Department of Health, 201 W. Preston Street, Baltimore, MD 21201 USA; 5 MedStar Center for Biostatistics, Informatics and Data Science, MedStar Health Research Institute, 3007 Tilden St.Suite 6N, Washington, NWDC 20008 USA; 6 Department of Oncology, Georgetown University School of Medicine, 3900 Reservoir Road, Washington, NWDC 20007 USA

**Keywords:** Social needs screening, Demographic data collection, Primary care, Community resources

## Abstract

**Background:**

Health outcomes are strongly impacted by social determinants of health, including social risk factors and patient demographics, due to structural inequities and discrimination. Primary care is viewed as a potential medical setting to assess and address individual health-related social needs and to collect detailed patient demographics to assess and advance health equity, but limited literature evaluates such processes.

**Methods:**

We conducted an analysis of cross-sectional survey data collected from *n* = 507 Maryland Primary Care Program (MDPCP) practices through Care Transformation Requirements (CTR) reporting in 2022. Descriptive statistics were used to summarize practice responses on social needs screening and demographic data collection. A stepwise regression analysis was conducted to determine factors predicting screening of all vs. a targeted subset of beneficiaries for unmet social needs.

**Results:**

Almost all practices (99%) reported conducting some form of social needs screening and demographic data collection. Practices reported variation in what screening tools or demographic questions were employed, frequency of screening, and how information was used. More than 75% of practices reported prioritizing transportation, food insecurity, housing instability, financial resource strain, and social isolation.

**Conclusions:**

Within the MDPCP program there was widespread implementation of social needs screenings and demographic data collection. However, there was room for additional supports in addressing some challenging social needs and increasing detailed demographics. Further research is needed to understand any adjustments to clinical care in response to identified social needs or application of data for uses such as assessing progress towards health equity and the subsequent impact on clinical care and health outcomes**.**

**Supplementary Information:**

The online version contains supplementary material available at 10.1186/s12913-024-10948-7.

## Background

There is increasing attention on the impact of factors such as economic stability, education, neighborhood, and built environment on healthcare outcomes and, in particular, how primary care settings can assess and address individual level health-related social needs (HRSN) [1, 2]. In turn, the American Academy of Pediatrics (AAP) and the American Academy of Family Physicians (AAFP) both recommend that primary care providers screen and address social needs as part of routine primary care visits [3]. Patients with unmet social needs are at a higher risk of missing appointments, frequent emergency room visits, and hospitalization and rehospitalization [4, 5]. Identifying social needs and collecting detailed patient demographics in primary care can be used to tailor care, allocate resources effectively, and advocate for equitable policies, making these workflows a critical step towards advancing health equity [1–3].

Despite acknowledgement of the importance of integrating social care in clinical settings including a recent mandate by the Centers for Medicare and Medicaid services for screening in inpatient settings, the implementation of social needs screening and demographic data collection is complex and resource intensive [6, 7]. Furthermore, patients who screen positive for social needs may decline assistance to address those needs. These occurrences may prove frustrating to those conducting screening if they lack sufficient training on delivering screening or assisting individuals with addressing social needs [8]. Additionally, while many practices already collect basic demographic data such as age, ethnicity, and race, demographic information is not always collected in a culturally sensitive or inclusive manner. Demographic data collection processes are not standardized, and many demographic fields (e.g., education level, sexual orientation, and disability status) are sometimes not asked at all. As part of a contract to provide technical assistance to Maryland Primary Care Program (MDPCP) practices to support social needs screening and demographic data collection, we explored collected survey data to understand current practices around social needs screening and demographic data collection as well as potential areas for growth in screening delivery.

## Methods

### Study population

MDPCP is a voluntary program for eligible primary care practices that provides funding and support for the delivery of advanced primary care for Medicare beneficiaries throughout Maryland. MDPCP supports the overall health care transformation process and allows primary care providers to play an increased role in disease prevention, management of chronic disease, and prevention of unnecessary hospital utilization [9]. The primary goal of MDPCP is the sustainable transformation of primary care across Maryland to include all the elements of advanced primary care to support the health needs of state residents [9]. MDPCP is co-administered by teams at the Maryland Department of Health and the Center for Medicare and Medicaid Innovation (CMMI). At the time of the survey, the MDPCP network included *n* = 507 participating primary care practices representative of every county in Maryland.

MDPCP offers a combination of financial incentives and other supports tailored to primary care practices. These incentives encompass non-visit-based payments specifically designed for care coordination initiatives, as well as performance-based incentives, rewarding practices for achieving clinical quality, patient experience, and utilization benchmarks. In addition to financial incentives, MDPCP provides a variety of additional supports for care transformation MDPCP practices are paired with a Practice Transformation Coaches, who provide guidance, answer questions, and work directly with practices to improve processes that improve quality of care and decrease costs. In addition to Coaches, practices have access to the MDPCP Learning System encompassing a myriad of learning opportunities including User Groups, All-Practice Calls, and other collaborative forums for practices to learn from subject matter experts and fellow participants. Practices also have access to a handful of Guides including the Advancing Primary Care Guide, which provides information on MDPCP requirements, tactics for advancing the functions of primary care, and achieving care transformation. Additionally, practices have the option to partner with a Care Transformation Organization (CTO), who can assist with care management or other related patient services.

### Data collection

Care transformation requirement (CTR) reporting questions ask MDPCP participants about progress on specific MDPCP requirements that span the five comprehensive primary care functions (Appendix 1). The five key functions of advanced primary care are care management, access and continuity, comprehensiveness, and coordination across the continuum of care, beneficiary and caregiver experience, and planned care for health outcomes. The questionnaire is developed by CMMI, and MDPCP participants respond in the online Centers for Medicare and Medicaid Services (CMS) program portal twice annually, as a requirement of program participation (Appendix 2). The survey used in this analysis was collected in the third quarter of 2022. This analysis was deemed exempt by the Georgetown/MedStar Institutional Review Board (Study 4698).

### Statistical analysis

We used descriptive statistics to review social needs screening and demographic data collection responses from MDPCP practices*.* We conducted additional analysis to investigate responses by practice characteristics including practice size (small 1–2, medium 3–7, large 8 + providers) and hospital affiliation (yes or no). Further, a stepwise regression analysis was used to determine factors predicting the routine screening of beneficiaries for unmet social needs, comparing all beneficiaries to a specific targeted subsection. Variables used in the model were practice size, and hospital affiliation. 487 of the 507 records were used for regression analyses. We excluded practices if they did not report screening beneficiaries (*n* = 4), practice size (*n* = 1), or hospital affiliation status (*n* = 15). SAS 9.4 (Cary, NC) was used in all analyses.

## Results

Practice responses on social needs screening and referral processes are presented in Table 1. Among the MDPCP practices, nearly all reported some form of social needs screening for all (63%) or at least some (36%) beneficiaries. Many practices reported utilizing a social needs screening tool developed by the practice or affiliated health system (32%). Other practices reported screening using standardized screening tools, including, an unspecified standardized tool (21%); EHR-specific tool (19%); Accountable Health Communities (14%); and PRAPARE (5%). There was substantial variation in EHR vendors, with 23% of practices using EPIC, 17% using eClinicalWorks, 14% using Cerner, and 11% using Athenahealth. Approximately half (49.5%) of the practices reported conducting social needs screening annually, while 18% of practices reported conducting screenings at every visit and 15% when indicated based on reason for visit. Just over a quarter (27%) of practices reported linking responses to discrete ICD-10 or Social Determinants of Health (SDOH) Z codes.

**Table 1:** Social needs screening and demographic data collection reported by practices

**Do you routinely screen your beneficiaries for unmet social needs?**	**N**	**%**
All beneficiaries	317	62.5
Targeted Subpopulation	186	36.4
Do not screen	4	0.8
**What screener do you use?**	**N**	**%**
Tool Developed by Practice or System	214	31.6
Other Standardized Screening Tool	139	20.5
Tool Developed by EHR	132	19.5
AHC	96	14.2
PRAPARE	31	4.6
Your Current Life Situation (Kaiser)	3	0.4
Other	63	9.3
**How often do you screen your beneficiaries for unmet social needs**	**N**	**%**
Annually	249	49.5
At Every Visit	89	17.7
When indicated based on reason for visit	77	15.3
Other	73	14.5
Twice per year	12	2.4
Only at their initial visit	3	0.6
**Are screening tools or questions integrated with your EHR or health IT system?**	**N**	**%**
Yes	420	83.5
No	83.0	16.5
**Does screening data link to discrete ICD-10 Z-codes/diagnosis code information?**	**N**	**%**
Yes	115	27.4
No	305	72.6
**Do you routinely collect patient demographics from your beneficiaries?**	**N**	**%**
We collect patient demographics from all Beneficiaries	504	99.4
We collect patient demographics from some Beneficiaries	3	0.6
**Demographic Questions are asked by a support staff member**	**N**	**%**
Yes	355	70.0
No	152	30.0
**How often is patient demographic information collected?**	**N**	**%**
Annually	116	22.9
At Every Visit	258	50.9
Only at their initial visit	102	20.1
Twice per year	14	2.8
Other	17	3.4
**Is patient demographic information integrated with your EHR or health IT system?**	**N**	**%**
No	4	0.8
Yes, all	479	94.5
Yes, some	24	4.7

Survey responses revealed variability regarding which patients receive social needs screening, screening frequency, EHR integration and use of Z-codes based on practice characteristics (Appendix 3). In an exploratory multivariate logistic regression we found that practices with a hospital affiliation were more likely to screen a targeted population than all patients (OR = 1.54, 95% CI = 1.05–2.27) and practices that were small- (1–2 providers) or medium-sized (3–7 providers) were more likely to screen all patients. (OR = 0.46, 95% CI = 0.26–0.80; OR = 0.46, 95% CI = 0.27–0.78, respectively; data shown in text only). Practices had the opportunity to describe which beneficiaries were targeted. Responses included individuals at high risk (*n* = 67) or experiencing recent mental or clinical health events (*n* = 18), participants in care management or care coordination programs (*n* = 82), Health Equity Advancement Resource and Transformation (HEART) patients (*n* = 25), and attendees of annual wellness visits (*n* = 40).

When practices were asked to select social needs that they prioritize, common responses were transportation (93%), food insecurity (89%), housing instability (86%), financial resource strain (85%), and social isolation (84%) (Table 2). The least common needs prioritized were internet access (42%), phone access (46%), employment (48%), and language access (51%). Practices also reported which social needs were most challenging to support. The greatest challenges came with addressing housing instability (31%), internet access (31%), financial resource strain (30%), and medication affordability (30%).

**Table 2:** Social needs prioritized among MDPCP practices and challenges connecting beneficiaries with resources to address this need

	*The % of practices that prioritized the social need*	*The % of practices that experienced challenges with resources*
Transportation	92.5	27.7
Food Insecurity	88.8	24.0
Housing Instability	86.2	31.4
Financial Resource Strain	85.0	30.4
Social Isolation	83.8	26.1
Safety or Interpersonal Violence	72.0	20.3
Medication Affordability	68.0	29.9
Utility Needs	67.7	21.9
Lack of adequate insurance coverage	60.4	27.8
Language Access	51.3	18.1
Employment	47.9	16.9
Phone Access	45.6	26.4
Internet Access	42.2	31.3

Nearly all practices reported collecting patient demographics in some capacity (99%), with most practices reporting that demographic data are collected by a staff member (70%), collected at every visit (51%), annually (23%), or only at the patient’s initial visit (20%). Race and primary language were collected by nearly all practices (96%), gender identity was collected by 92%, relationship status by 87%, ethnicity by 87%, and employment status by 84% of practices. Other demographic factors were less commonly asked: only 49% of practices reported asking about sexual orientation, 48% asked about disability status, and 38% asked about highest level of education.

## Discussion

In this study we found that primary care practices participating in the MDPCP program overall had a high rate of social risk factor screening, with many using screeners that had been developed to meet individual practice needs. Commonly prioritized domains included transportation, food insecurity, housing instability, financial strain, and social isolation, the last being a commonly cited problem among older adults. Describing patterns of screening and demographics in this sample of practices across the state increase understanding of successes and challenges in real-world practice settings and informs potential future interventions.

Determining which patients should be screened and by whom in a busy primary care setting, as well as who can respond to identified needs, can be challenging. In our study there were differences both in which patients were screened and how often by practice [10, 11]. Open ended responses suggested that among some MDPCP practices, screening was performed only for individuals who qualify for extra social assistance through the MDPCP program (i.e., those who qualify due to medical complexity and area deprivation index). Although we did not find other published literature focused specifically on Medicare patients at the state level, we found literature on programs focused on social needs screening among Medicaid populations in several states. Like Maryland practices, standardized measures and consistent approaches to measuring social needs have not been adopted or required in many states [12–14]. Further, a high percentage of the Maryland practices reported using home grown and standardized screening tools with additional questions to meet the practices’ needs. While the ability to aggregate social needs data across care settings can be challenging with different screeners, there is national movement to harmonize domains across various social risk factor screeners through the Gravity Project and the Office of the National Coordinator [12, 15]. Notably, CMS has mandated social needs reporting in the inpatient setting beginning January 2024 for five specific domains, but has not specified a single tool or set of tools given that while there are some validated subsets of questions (e.g., Hunger Vital Signs), there is currently no gold standard tool [16]. Potential hurdles in requiring specific tools may include limitations on EHR technology, referral processes, and provider or staff level comfort and training in asking specific questions. Furthermore, implementing screening without supports for training the staff on trauma-informed approaches and how to respond to identified needs has the potential to cause more harm than benefit to patients. Thus, toolkits established by various professional societies and public health societies may be useful to determine which tools are most appropriate for a given practice and how to integrate them into care where practices have not yet started screening or encounter challenges [17–19].

Regarding practices with a hospital affiliation being more likely to screen a targeted population, one possibility is that practices affiliated with hospitals may have access to additional resources and supports that facilitate targeted screening efforts. Hospitals often have established practices including social risk factor screening for targeted subpopulations to address costly hospital readmissions, which may encourage affiliated practices to deliver more targeted screening practices. While it is unclear why small or medium-sized practices were more likely to screen all patients than a sub-population, it may have to do with more autonomy in workflow process, less customization of the EHR to target sub-populations, or differences in staffing and provider to patient ratios. While we cannot explain these differences from the survey alone, findings suggest that the size and affiliation of practices play a role in their screening practices, highlighting the importance of considering practice characteristics when designing specific supportive interventions or policies aimed at increasing screening rates.

It is important to highlight that MDPCP practices have achieved impressive levels of social needs screening and demographic data collection implementation. This success could be attributed largely to the program’s requirements and incentives to screen beneficiaries for social needs and collect demographic information. Additionally, the program provides technical support and resources to meet these requirements and to stand up social needs screening workflows if not already in place. By joining MDPCP, participating practices have demonstrated a commitment to advanced primary care, further indicating MDPCP participation may be associated with higher uptake of these workflows, as opposed to primary care practices who do not participate in similar value-based programs. Other states considering such programs may look to some of these supports when rolling out new requirements or incentives.

While the findings highlight the high level of social needs screening and demographic data collection, challenges in addressing identified needs may also be due to various factors including complexity of workflows and staffing, patients with social needs declining assistance, or limited local resource availability [20]. Previous research suggests patients may decline social needs assistance in healthcare settings if they do not feel like they need help, are confused about what is offered, are not confident that the assistance would be helpful, have experienced previous negative experiences, or feel fear and mistrust related to disclosing personal information [8]. In areas that posed the greatest referral challenges, policy efforts may be needed to deliver services and bridge the gaps to access. For example, the challenge of addressing housing needs is not newly identified; previous literature has shown increasing costs and declining supply have contributed to national housing availability and affordability challenges [21, 22]. Medication cost continues to be a major problem cited in the literature, especially for older populations with a higher incidence of chronic diseases [23, 24]. Financial strain among individuals often poses a challenge as financial needs fluctuate frequently, and changes can be dramatic; further, these changing needs over time are often not resolved by a one-time intervention and require long-term involvements [11]. Though research on the effects of internet access and health outcomes is still emerging, literature suggests investment in digital infrastructure by federal, state, and local governments is needed for further development of the internet as a means of addressing long-standing inequality in health [25, 26]. While food insecurity and transportation were top needs prioritized within MDPCP practices, they did not present the same level of challenge to practices, perhaps due to wider availability of resources, partnerships, and supports such as transportation vouchers.

Although addressing connection to resources continues to be a challenge for practices, there are opportunities to leverage information from social needs screenings and demographic data collection in several other ways to improve care. Aggregate screening and demographic data can be used for quality improvement initiatives within primary care practices by analyzing trends and patterns in social needs data to help practices identify areas of unmet need, track outcomes, and update protocols for screening and referral processes. Additionally, data can be used to advocate for policy changes to address systemic issues affecting patients’ health outcomes. However, challenges in utilizing information from social needs screening and demographic data collection may still exist due to limited resources and capacity and lack of provider awareness and training availability.

Increased collection of detailed demographic data, particularly regarding sexual orientation, education level, and disability status presents an opportunity for improvement in primary care. Furthermore, collecting detailed demographic information can better allow practices to understand the need for targeted educational materials, track quality indicators, and address challenges faced by historically marginalized populations [26, 27]. Still, even with good data collection approaches, some practices do not have the infrastructure or resources to analyze data to assess disparities in care or outcomes.

This study's strengths lie in its comprehensive analysis of a diverse range of primary care practices across Maryland. The inclusion of 507 practices with variations in size, location, and demographics enhances the representativeness of the findings and improves the generalizability of the results to a broader population. Consequently, the findings derived from studying a large population can contribute to a stronger evidence base for decision-making in healthcare and support the development of effective interventions and policies. A limitation of the study is the reliance on self-report, which may depend on the participants’ perspectives. Additionally, MDPCP practices meet eligibility criteria and voluntarily select to join the program, so these practices may be better equipped to join a value-based program that includes requirements or incentives to screen for social needs. Despite the limitations, our findings are novel in that few published studies highlight current practices at scale on social risk factor screening and referral in outpatient primary care settings for adults. Future research is warranted to show what strategies effectively increase uptake and drive meaningful change in social-needs responsive healthcare delivery.

## Conclusion

MDPCP practices have demonstrated widespread adoption of social risk factor screenings and needs prioritization. While practices have implemented strategies to link patients to resources to address needs, challenges remain with providing social needs resources to beneficiaries from the primary care setting. Additionally, there is room for improvement in collecting certain demographic data fields within primary care practices. As the present analysis was based on cross-sectional data, future studies are needed to understand how to effect change in implementing or scaling social risk factor screening and detailed demographic data collection at the practice level. Additionally, future work is needed to understand how care is adjusted in response to identified social needs and how that impacts outcomes at the patient level.

### Supplementary Information

**Supplementary Material 1.**

**Supplementary Material 2.**

**Supplementary Material 3.**

## Data Availability

To access the datasets examined in this study, interested parties must follow the procedure outlined by the CMS. Requests should be submitted through the CMS website (cms.gov), and any queries can be directed to FOIA_request@cms.hhs.gov.

## Abbreviations

MDPCP: Maryland Primary Care Program
CTR: Care Transformation Requirements
CMMI: Center for Medicare and Medicaid Innovation
CMS: Centers for Medicare and Medicaid Services
EHR: Electronic Health Record
SDOH: Social Determinants of Health
