# Supplementary material for: Understanding social needs screening and demographic data collection in primary care practices serving Maryland Medicare patients
Source: BMC Health Serv Res. 2024 Apr 10;24:448. doi: 10.1186/s12913-024-10948-7 (PMC11005183; doi:10.1186/s12913-024-10948-7)
Supplement: Supplementary file 1 — Supplementary Material 1. [file 12913_2024_10948_MOESM1_ESM.docx]

**Appendix 1**

CTR requirements

| Comprehensive Primary Care Functions of Advanced Primary Care | Care Transformation Requirement |
| --- | --- |
| Access and Continuity | 1.1 Empanel attributed beneficiaries to practitioner or care team  1.2 Ensure attributed beneficiaries have 24/7 access to a care team or practitioner with real-time access to the EHR.  3 Ensure attributed beneficiaries have regular access to the care team or practitioner through at least one alternative care strategy |
| Care Management | 2.1 Ensure all empaneled, attributed beneficiaries are risk stratified.  2.2 Ensure all attributed beneficiaries identified as increased risk and likely to benefit receive targeted, proactive, relationship-based (longitudinal) care management.  2.3 Ensure attributed beneficiaries receive a follow-up interaction from your practice within one week for ED discharges and two business days for hospital discharges.  2.4 Ensure targeted, attributed beneficiaries who have received follow-up after ED, hospital discharge, or other triggering events receive short-term (episodic) care management.  2.5 Ensure attributed beneficiaries in longitudinal care management are engaged in a personalized care planning process, which includes at least their goals, needs, and self-management activities.  2.6 Ensure attributed beneficiaries in longitudinal care management have access to comprehensive medication management. |
| Comprehensiveness and Coordination across the Continuum of Care | 3.1 Ensure coordinated referral management for attributed beneficiaries seeking care from high-volume and/or high-cost specialists as well as EDs and hospitals.  3.2 Ensure attributed beneficiaries with behavioral health needs have access to care consistent with at least one option from a menu of options for integrated behavioral health supplied to attributed beneficiaries by the Practice.  3.3 Facilitate access to resources that are available in your community for beneficiaries with identified health-related social needs. |
| Beneficiary & Caregiver Experience | 4.1 Convene a Patient-Family/ Caregiver Advisory Council (PFAC) at least annually and integrate PFAC recommendations into care and quality improvement activities.  4.2 Engage attributed beneficiaries and caregivers in a collaborative process for advance care planning |
| Planned Care for Health Outcomes | 5.1 Continuously improve your performance on key outcomes, including cost of care, electronic clinical quality measures, beneficiary experience, and utilization measures. |
