# Supplementary material for: Understanding social needs screening and demographic data collection in primary care practices serving Maryland Medicare patients
Source: BMC Health Serv Res. 2024 Apr 10;24:448. doi: 10.1186/s12913-024-10948-7 (PMC11005183; doi:10.1186/s12913-024-10948-7)
Supplement: Supplementary file 2 — Supplementary Material 2. [file 12913_2024_10948_MOESM2_ESM.docx]

**Appendix 2.** Care Transformation Reporting on Linkages with Social Services Questionnaire

| **Do you routinely screen your beneficiaries for unmet social needs?**   - We do not screen beneficiaries for unmet social needs - We screen a targeted subpopulation of beneficiaries for unmet social needs   - Please specify the targeted subpopulation(s) and why you chose to target this group: (Open text) - We universally screen all beneficiaries for unmet social needs |
| --- |
| **How often do you screen your beneficiaries for unmet social needs?**   - We do not screen beneficiaries for unmet social needs - At every visit - Twice per year - Annually - Only at their initial visit - When indicated based on reason for visit - Other (please specify) |
| **What type of screening tool(s) do you use or adopt to capture unmet social needs in your beneficiary population? (Select all that apply)**   - We do not use any screening tools - Accountable Health Communities (AHC) tool - PRAPARE (National Association of Community Health Centers) tool - Your Current Life Situation (Kaiser Permanente) tool - Other Standardized screening tool (e.g., screening tools published by HealthLeads, IOM/NAM)   - Please specify the standardized screening tool that your practice uses (Open text) - Tool developed by EHR (e.g., Epic SDOH tool) company - Tool developed by practice or health system - Other, please specify (Text Field) |
| Are screening tools or questions integrated with your EHR or health IT system?   - Yes - No |
| Does screening data link to discrete ICD-10 Z-codes/diagnosis code information?   - Yes - No |
| What are the health-related social needs your practice has prioritized to address in your beneficiary population? (Select all that apply)   - We have not prioritized any social needs to address in our beneficiary population  \| Health-Related Social Needs \| Do you have an established, ongoing relationship with social resources to address this need? \| Do you have challenges connecting beneficiaries with resources to address this need? \| \| --- \| --- \| --- \| \| - Food insecurity \| - Yes - No \| - Yes - No \| \| - Housing instability \| - Yes - No \| - Yes - No \| \| - Utility needs \| - Yes - No \| - Yes - No \| \| - Financial resource strain \| - Yes - No \| - Yes - No \|  \| - Medication affordability \| - Yes - No \| - Yes - No \| \| --- \| --- \| --- \| \| - Transportation \| - Yes - No \| - Yes - No \| \| - Employment \| - Yes - No \| - Yes - No \| \| - Social isolation \| - Yes - No \| - Yes - No \| \| - Safety or interpersonal violence \| - Yes - No \| - Yes - No \|  \| - Phone access \| - Yes - No \| - Yes - No \| \| --- \| --- \| --- \| \| - Internet access \| - Yes - No \| - Yes - No \|  \| - Language access \| - Yes - No \| - Yes - No \| \| --- \| --- \| --- \|  \| - Lack of adequate insurance coverage \| - Yes - No \| - Yes - No \| \| --- \| --- \| --- \| \| - Other, please specify (Text Field) \| - Yes - No \| - Yes - No \| |
| Do you have an inventory of social service resources?   - Yes - No |
| How frequently is the inventory of social service resources your practice uses updated?   - Ad hoc basis only - At least monthly - Every 2-6 months - Every 6-12 months - Less than annually |
| Do you confirm when patients follow up with social service referrals or receive any information back about the social services referrals?   - Yes - No - Sometimes (please specify) |
| Describe any barriers to prioritizing health-related social needs. (Optional) (Text Area) |
| Do you routinely collect patient demographics from your beneficiaries?   - We do not collect patient demographics from our beneficiaries - We collect patient demographics from some of our beneficiaries   - Please specify the population of beneficiaries chosen and why you chose to target this group: (Open text) - We collect patient demographics from all beneficiaries |
| What patient demographics do you collect from your beneficiaries?   - Gender identify - Race - Ethnicity - Primary Language - Sexual orientation - Education - Relationship status - Employment status - Disability status - Other (please specify) |
| How are patient demographics collected? Select all that apply.   - Demographic questions are asked by a support staff member or provider - Patients fill out information on paper or in - Patients fill out in computer directly or through patient portal - Other method: (please specify) |
| How often is patient demographic information collected?   - We do not collect patient demographics from beneficiaries - At every visit - Twice per year - Annually - Only at their initial visit - Other (please specify) |
| Is patient demographic information integrated with your EHR or health IT system?   - Yes, all - Yes, some - No, not integrated |
