# Supplementary material for: Understanding social needs screening and demographic data collection in primary care practices serving Maryland Medicare patients
Source: BMC Health Serv Res. 2024 Apr 10;24:448. doi: 10.1186/s12913-024-10948-7 (PMC11005183; doi:10.1186/s12913-024-10948-7)
Supplement: Supplementary file 3 — Supplementary Material 3. [file 12913_2024_10948_MOESM3_ESM.docx]

**Social needs screening and demographic data collection reported by practice characteristics**

|  | **Small** | | **Med** | | **Large** | |  | **No Hospital Affiliation** | | **Hospital Affiliation** | |  |
| --- | --- | --- | --- | --- | --- | --- | --- | --- | --- | --- | --- | --- |
|  | N | % | N | % | N | % | p-value | N | % | N | % | p-value |
| **Do you routinely screen your beneficiaries for unmet social needs?** | | | |  |  |  |  |  |  |  |  |  |
| All Beneficiaries | 135 | 67.2 | 146 | 63.5 | 35 | 46.7 | 0.0104 | 180 | 66.4 | 124 | 56.6 | 0.0169 |
| Do Not Screen | 3 | 1.5 | 1 | 0.4 | 0 | 0.0 |  | 3 | 1.1 | 0 | 0.0 |  |
| Targeted Subpopulation | 63 | 31.3 | 83 | 36.1 | 40 | 53.3 |  | 88 | 32.5 | 95 | 43.4 |  |
| **What screener do you use?** |  |  |  |  |  |  |  |  |  |  |  |  |
| PRAPARE | 15 | 7.5 | 13 | 5.7 | 3 | 4.0 |  | 31 | 11.4 | 0 | 0.0 |  |
| AHC | 39 | 19.4 | 46 | 20.0 | 11 | 14.7 |  | 54 | 19.9 | 40 | 18.3 |  |
| Your Current Life Situation (Kaiser) | 3 | 1.5 | 0 | 0.0 | 0 | 0.0 |  | 3 | 1.1 | 0 | 0.0 |  |
| Other Standardized Screening Tool | 53 | 26.4 | 69 | 30.0 | 17 | 22.7 |  | 56 | 20.7 | 79 | 36.1 |  |
| Tool Developed by EHR | 39 | 19.4 | 59 | 25.7 | 33 | 44.0 |  | 46 | 17.0 | 82 | 37.4 |  |
| Tool Developed by Practice or System | 98 | 48.8 | 95 | 41.3 | 20 | 26.7 |  | 125 | 46.1 | 80 | 36.5 |  |
| Other | 18 | 9.0 | 28 | 12.2 | 17 | 22.7 |  | 48 | 17.7 | 15 | 6.8 |  |
| **How often do you screen your beneficiaries for unmet social need** | | | | | | | | | | | | |
| Annually | 101 | 50.2 | 119 | 51.7 | 29 | 38.7 | <.0001 | 143 | 52.8 | 98 | 44.7 | 0.0102 |
| At Every Visit | 40 | 19.9 | 37 | 16.1 | 11 | 14.7 |  | 44 | 16.2 | 39 | 17.8 |  |
| Other | 7 | 3.5 | 41 | 17.8 | 25 | 33.3 |  | 30 | 11.1 | 43 | 19.6 |  |
| Twice Per Year | 8 | 4.0 | 4 | 1.7 | 0 | 0.0 |  | 11 | 4.1 | 1 | 0.5 |  |
| When Indicated Based On Reason For Visit | 41 | 20.4 | 26 | 11.3 | 10 | 13.3 |  | 39 | 14.4 | 36 | 16.4 |  |
| Only At Their Initial Visit | 1 | 0.5 | 2 | 0.9 | 0 | 0.0 |  | 1 | 0.4 | 2 | 0.9 |  |
| **Are screening tools or questions integrated with your EHR or health IT system?** | | | | | |  |  |  |  |  |  |  |
| Yes | 152 | 75.6 | 202 | 87.8 | 65 | 86.7 | 0.0047 | 193 | 71.2 | 215 | 98.2 | <0.0001 |
| No | 46 | 22.9 | 27 | 11.7 | 10 | 13.3 |  | 75 | 27.7 | 4 | 1.8 |  |
| **Does screening data link to discrete ICD-10 Z-codes/diagnosis code information?** | | | | | |  |  |  |  |  |  |  |
| Yes | 59 | 38.8 | 38 | 18.9 | 17 | 22.7 | 0.0002 | 76 | 39.4 | 36 | 16.7 | <0.0001 |
| No | 93 | 61.2 | 164 | 81.6 | 58 | 77.3 |  | 117 | 60.6 | 179 | 83.3 |  |
| **Do you routinely collect patient demographics from your beneficiaries?** | | | | |  |  |  |  |  |  |  |  |
| We Collect Patient Demographics From All Beneficiaries | 199 | 99.0 | 229 | 99.6 | 75 | 100.0 | 0.5780 | 270 | 99.6 | 217 | 99.1 | 0.4426 |
| We Collect Patient Demographics From Some Beneficiaries | 2 | 1.0 | 1 | 0.4 | 0 | 0.0 |  | 1 | 0.4 | 2 | 0.9 |  |
| **Demographic data collected** |  |  |  |  |  |  |  |  |  |  |  |  |
| Gender Identity | 180 | 89.6 | 219 | 95.2 | 68 | 90.7 |  | 247 | 91.1 | 205 | 93.6 |  |
| Race | 185 | 92.0 | 226 | 98.3 | 75 | 100.0 |  | 262 | 96.7 | 208 | 95.0 |  |
| Ethnicity | 165 | 82.1 | 205 | 89.1 | 68 | 90.7 |  | 229 | 84.5 | 197 | 90.0 |  |
| Primary Language | 189 | 94.0 | 225 | 97.8 | 71 | 94.7 |  | 262 | 96.7 | 208 | 95.0 |  |
| Sexual Orientation | 96 | 47.8 | 113 | 49.1 | 40 | 53.3 |  | 140 | 51.7 | 103 | 47.0 |  |
| Education | 87 | 43.3 | 77 | 33.5 | 26 | 34.7 |  | 107 | 39.5 | 83 | 37.9 |  |
| Relationship Status | 169 | 84.1 | 207 | 90.0 | 66 | 88.0 |  | 230 | 84.9 | 202 | 92.2 |  |
| Employment Status | 165 | 82.1 | 195 | 84.8 | 66 | 88.0 |  | 224 | 82.7 | 193 | 88.1 |  |
| Disability Status | 106 | 52.7 | 101 | 43.9 | 35 | 46.7 |  | 128 | 47.2 | 108 | 49.3 |  |
| Other | 3 | 1.5 | 19 | 8.3 | 13 | 17.3 |  | 46 | 17.0 | 15 | 6.8 |  |
| **Demographic Questions are asked by a support staff member** | | | |  |  |  |  |  |  |  |  |  |
| Yes | 125 | 62.2 | 168 | 73.0 | 61 | 81.3 | 0.0033 | 197 | 72.7 | 147 | 67.1 | 0.1801 |
| No | 76 | 37.8 | 62 | 27.0 | 14 | 18.7 |  | 74 | 27.3 | 72 | 32.9 |  |
| **How often is patient demographic information collected?** | | |  |  |  |  |  |  |  |  |  |  |
| Annually | 62 | 30.8 | 44 | 19.1 | 10 | 13.3 | <.0001 | 52 | 19.2 | 60 | 27.4 | 0.0101 |
| At Every Visit | 70 | 34.8 | 138 | 60.0 | 50 | 66.7 |  | 138 | 50.9 | 113 | 51.6 |  |
| Only At Their Initial Visit | 50 | 24.9 | 39 | 17.0 | 13 | 17.3 |  | 67 | 24.7 | 29 | 13.2 |  |
| Other | 7 | 3.5 | 8 | 3.5 | 2 | 2.7 |  | 7 | 2.6 | 10 | 4.6 |  |
| Twice Per Year | 12 | 6.0 | 1 | 0.4 | 0 | 0.0 |  | 7 | 2.6 | 7 | 3.2 |  |
| **Is patient demographic information integrated with your EHR or health IT system?** | | | | | | |  |  |  |  |  |  |
| No | 3 | 1.5 | 1 | 0.4 | 0 | 0.0 | 0.0056 | 1 | 0.4 | 3 | 1.4 | 0.4038 |
| Yes, All | 181 | 90.0 | 226 | 98.3 | 71 | 94.7 |  | 258 | 95.2 | 204 | 93.2 |  |
| Yes, Some | 17 | 8.5 | 3 | 1.3 | 4 | 5.3 |  | 12 | 4.4 | 12 | 5.5 |  |
